# Perception of Medication Safety–Related Behaviors Among Different Age Groups: Web-Based Cross-Sectional Study

**DOI:** 10.2196/58635

**Published:** 2024-08-12

**Authors:** Yan Lang, Kay-Yut Chen, Yuan Zhou, Ludmila Kosmari, Kathryn Daniel, Ayse Gurses, Richard Young, Alicia Arbaje, Yan Xiao

**Affiliations:** 1 Department of Business State University of New York at Oneonta Oneonta, NY United States; 2 College of Business University of Texas at Arlington Arlington, TX United States; 3 Department of Industrial, Manufacturing, and Systems Engineering University of Texas at Arlington Arlington, TX United States; 4 College of Nursing and Health Innovation University of Texas at Arlington Arlington, TX United States; 5 School of Medicine Johns Hopkins University Baltimore, MD United States; 6 Family Medicine Residency Program The John Peter Smith (JPS) Health Network Fort Worth, TX United States; 7 Department of Health Policy and Management Johns Hopkins Bloomberg School of Public Health Baltimore, MD United States

**Keywords:** medication safety, patient engagement, aged adults, survey, Amazon Mechanical Turk, medication, engagement, older adults, elderly, safety, United States, USA, crowdsourcing, community, patient portal, primary care, medications, safety behavior, younger adults, age, correlation, statistical test

## Abstract

**Background:**

Previous research and safety advocacy groups have proposed various behaviors for older adults to actively engage in medication safety. However, little is known about how older adults perceive the importance and reasonableness of these behaviors in ambulatory settings.

**Objective:**

This study aimed to assess older adults’ perceptions of the importance and reasonableness of 8 medication safety behaviors in ambulatory settings and compare their responses with those of younger adults.

**Methods:**

We conducted a survey of 1222 adults in the United States using crowdsourcing to evaluate patient behaviors that may enhance medication safety in community settings. A total of 8 safety behaviors were identified based on the literature, such as bringing medications to office visits, confirming medications at home, managing medication refills, using patient portals, organizing medications, checking medications, getting help, and knowing medications. Respondents were asked about their perception of the importance and reasonableness of these behaviors on a 5-point Likert rating scale in the context of collaboration with primary care providers. We assessed the relative ranking of behaviors in terms of importance and reasonableness and examined the association between these dimensions across age groups using statistical tests.

**Results:**

Of 1222 adult participants, 125 (10.2%) were aged 65 years or older. Most participants were White, college-educated, and had chronic conditions. Older adults rated all 8 behaviors significantly higher in both importance and reasonableness than did younger adults (*P*<.001 for combined behaviors). Confirming medications ranked highest in importance (mean score=3.78) for both age groups while knowing medications ranked highest in reasonableness (mean score=3.68). Using patient portals was ranked lowest in importance (mean score=3.53) and reasonableness (mean score=3.49). There was a significant correlation between the perceived importance and reasonableness of the identified behaviors, with coefficients ranging from 0.436 to 0.543 (all *P*<.001).

**Conclusions:**

Older adults perceived the identified safety behaviors as more important and reasonable than younger adults. However, both age groups considered a behavior highly recommended by professionals as the least important and reasonable. Patient engagement strategies, common and specific to age groups, should be considered to improve medication safety in ambulatory settings.

## Introduction

Engagement of older adult patients has been recognized as key to health outcomes including safety [1]. While specific skills and attitudes related to patient engagement have been identified and measured [2,3], there is a lack of clarity regarding the specific roles and responsibilities expected of patients in community settings, where patients and families are responsible for the medication use process. Health care organizations often set implicit expectations regarding the roles and responsibilities of patients and their families in collaborative activities such as planning, implementation, and discourse about their health [4].

The purpose of our study was to use a crowdsourcing approach to investigate individuals’ perceptions of the importance and reasonableness of medication safety behaviors across various age groups. We chose patient portal use as a reference for patient engagement behaviors due to extensive efforts by health care organizations and regulators to encourage this behavior. A survey conducted in 2020 showed that more than half of individuals nationwide were offered access to patient portals, with nearly 40% accessing their records [5]. Patients’ perspectives on the importance and reasonableness of using portals are important to understand in order to devise interventions to encourage the behavior, such as patients’ interest, willingness, and ability [6-8], especially among older adults [9].

Health care professionals are encouraged to guide patients and their families to actively participate in their care by adopting safety behaviors, although little is known about older adults’ perspectives on these behaviors and roles within the collaborative process to improve patient engagement in medication safety. By understanding the perspectives of laypeople, we can identify the gaps in engaging patients and family members in medication safety improvements and design interventions that can better meet their needs. Furthermore, understanding the relation between importance and reasonableness in perceiving medication safety behaviors is crucial for health care professionals to tailor their interventions and communication strategies effectively, particularly for older adults, to promote safer medication practices.

The aim of this study is to assess how older adults perceive the importance and reasonableness of 8 medication safety behaviors in ambulatory settings and to compare their responses with those of younger adults.

## Methods

### Study Design

This cross-sectional study was conducted using a role-playing survey to assess the importance and reasonableness of medication safety behaviors.

### Setting

The study was conducted using Amazon Mechanical Turk (MTurk), a crowdsourcing platform, from October to December 2022. Participants completed the survey online. This approach allowed us to efficiently gather data from a large group of participants [10].

### Participants

Our study limited participants to US adults (≥18 years old) who had established a strong reputation on MTurk, defined as completed 100+ tasks with at least 95% approval ratings [11]. This choice leverages the acknowledged representativeness of US MTurk samples for diverse psychological dimensions [12] while ensuring engaged and reliable participation, as users with good reputations are generally more motivated and provide accurate data [13]. We used the Software Platform for Human Interaction Experiments (SoPHIE; SoPHIELabs) to administer the surveys. Participants were screened for eligibility through SoPHIE and were required to read and sign a consent form before participating. Qualified participants were given an online consent form, where they expressed their voluntary agreement to participate by clicking on the “Continue” button on their computer screen.

### Assessments

Participants were asked to envision themselves as older adults, retired individuals living alone with multiple health conditions (detailed instructions in Figure S1 in Multimedia Appendix 1). To identify safety behaviors in managing medication use in ambulatory settings, we reviewed literature and recommendations from safety organizations to represent professionals’ views on what patients should do to contribute to medication safety. For example, 1 study targeted behaviors associated with an office visit for patient engagement, including writing out a list of medications or bringing medications to visit [14]. In our survey, we defined the “importance” of a behavior as the extent to which all patients and families should adopt it for medication safety. “Reasonableness” was judged based on the assumption that following a treatment regimen makes sense if it leads to better health outcomes [15].

### Study Survey

This study used a carefully developed survey instrument. Initial pilot studies, with 14 closed-ended questions, assessed medication safety behaviors. Based on feedback from participants and experts, the survey was refined and consolidated for clarity and focus, resulting in a final 8-item instrument (Table 1). Throughout pilot testing, we iteratively evaluated the content validity of the questions against existing literature and organizational recommendations in medication safety. While patient involvement in developing the criteria and indicators was not direct, they were informed by a comprehensive review of relevant literature, safety advocacy group recommendations, and expert consensus in the field. Safe self-administration of medication heavily relies on patients’ knowledge about their treatments [16], their purpose, proper usage instructions, identifying and reporting adverse effects, obtaining refills, and effectively communicating any issues related to their prescribed medications with their health care provider. While the 8 behaviors specified in this study are not exhaustive, they encompass these crucial components and are presented for ease of understanding and application.

**Table 1:** Targeted patient behaviors in medication safety used in the survey.

Medication safety behaviors			Examples provided to participants		Justifications and references
**Bring medications**					
	Patients are expected to bring all medications and all relevant health-related documents to their health care provider office visits.	Collect all medicine bottles, including those over the counter such as Tylenol and vitamins, and bring them with you to the health care provider’s office. Make sure to also bring documents such as medication lists and blood sugar and blood pressure logs (if asked to keep one).		The FDA^a^ recommends keeping a list of all medications (prescribed and over the counter) and bringing it to all doctors’ appointments [17]. Although often encouraged by primary care providers [18,19]. Only about 20%-40% of the patients bring in their medications or medication list. Medication reviews lack standardization which can result in increased mortality, morbidity, and poor patient outcomes [20,21].	
**Confirm medications**					
	Patients will verify any changes in their medications after each provider’s visit.	You have been taking 20 mg of Simvastatin every day for cholesterol for a long time. In the last visit, your cholesterol level has decreased. Your provider reduced the medication dose to 10 mg. You make this change on your personal medication list.		FDA recommends to verify the medication list at least once a year or any time there is a change [17]. Medication discrepancies are very common among patients with chronic conditions, especially those who require frequent hospitalizations or see numerous providers [22,23].	
**Refill system**					
	Patients will establish a refill system.	Your provider advised you to call the pharmacy when you are about to run out of refills, and not their office. The pharmacy will contact the provider’s office for refill prescriptions. Using two 7-day pill boxes allows you to know 2 weeks in advance when a medicine will run out.		Current recommendations are to address all refill needs during the provider’s visit and send all prescriptions ideally to one pharmacy only in order to prevent gaps in medication therapies in chronic care due to disruptions and lapses in obtaining refills timely. Innovative systems use technological advances; however, older patients and individuals who speak English as a second language are less likely to use technology to refill medications [24-28].	
**Use portals**					
	Using patient health care portals.	Your provider’s office sent you a link for creating an account to access the patient portal website. After you sign up, you can use the portal to communicate with your provider and access your health information.		Patient portals were intended to improve the communication between the health care team and patients. They allow patients to be actively involved in their care, access their medical records, verify for accuracy, report concerns, and seek medical advice or medication refill [6,8,29-31].	
**Organize medications**					
	Using pill dispensers and other organizer tools.	Pill boxes are effective tools to remind you when and what medicines to take. You may also set reminders on your phone. To-go boxes are convenient to carry in your bag or purse when you are out, running errands. Charts, calendars, and electronic pill boxes are other ways of organizing medications.		The importance of having a system to organize medications was extensively studied. A list of memory tips and reminder systems (such as daily pillbox, calendar, or chart) to help organize scheduled prescriptions are available on various online resources [32-35].	
**Check medications**					
	Verifying medications for duplicates and expired medications.	The mail-order pharmacy sends you your refills automatically, so they always arrive before you run out of it. You know how to check the medicines against your list, as the color of the pills and names of medications (eg, generic vs brand name) may change from time to time, and you do not want to take duplicate medicines. You also dispose of expired medicines, so you do not accidentally take them.		The FDA and NIH^b^ recommend that patients check all medications for expiration dates. Ingesting expired medications may pose significant health hazards [17,36].	
**Medication awareness**					
	Accessing resources pertaining to medication-related issues.	You went to the pharmacy to pick up a prescription, but they did not have it. To clarify the situation, you call your provider’s office to inquire if you are still supposed to take the medication and verify the correct pharmacy on file. In another situation, you may need to call the pharmacist or the provider to find out what you need to do if you accidentally doubled your heart medicine.		The World Health Organization recommends that patients learn to identify and report any issues or side effects pertaining to taking medication [17,36].	
**Know medications**					
	Have basic knowledge about medications.	When you look at your medicine bottle, you are able to locate the medicine name, dose, when and how to take this medicine, how many refills are left, expiration date, and telephone number to call if you have questions about this medicine. For instance, you are prescribed to take a round white pill twice a day for high blood pressure. You wrote the name (“metoprolol”) on the medication list. You know to take 1 pill in the morning and 1 pill in the evening. You also know that you should take the pill with food. Symptoms to watch for are lightheadedness or very slow heartbeats.		Patients are advised to read carefully all information provided with the medications such as package inserts and pharmacy instructions. As many as an estimated 87% of patients do not read these instructions. Health illiteracy continues to be a challenge. Patients who are younger and have a higher formal education are more likely to have an adequate knowledge of medications [35,37-39].	

^a^FDA: US Food and Drug Administration.

^b^NIH: National Institutes of Health.

### Data Sources

Participants were asked to rate their perceptions of these behaviors in terms of importance and reasonableness on a 5-point Likert scale, with response categories of 1 (strongly disagree), 2 (disagree), 3 (neither agree nor disagree), 4 (agree), and 5 (strongly agree). After completing the survey, participants were asked to provide their demographic information (age, sex, race, ethnicity, education, and income) and number of chronic medical conditions.

### Study Size

The study size was determined by targeting a minimum of 1000 participants to ensure sufficient power to detect differences between age groups. This target was based on previous studies in the field that used similar methodologies and sample sizes to achieve robust statistical power and generalizability of findings [40,41]. We received 1222 completed surveys, achieving a completion rate of 94.5%, of the total 1293 attempts.

### Data Analysis

Statistical analyses were performed using Stata software (version 17; StataCorp). Differences in importance and reasonableness between 2 age groups (younger than 65 years and 65 years or older) were assessed by the Wilcoxon rank-sum tests. The associations between importance and reasonableness were assessed by the Pearson correlation coefficient. Disagreement was assessed by differences in ratings of reasonableness and importance scores for the same behavior by the same participant, with serious disagreement defined as a difference of 3 or more points. For ordered logistic regression models, the outcome variables were the perceived importance and reasonableness of the 8 medication safety behaviors, while the independent variables included age group, sex, ethnicity, education, income, and the number of chronic medical conditions. Each model used only 1 independent variable at a time to assess its individual impact on the outcome. Subgroup analyses with ordered logistic regression based on sex, ethnicity, education, income, and chronic medical conditions were conducted to understand how various factors influenced age-related perceptions of the 8 health behaviors.

### Ethical Considerations

#### Human Participant Ethics Review Approvals or Exemptions

This study was approved by the institutional review board (IRB) at the University of Texas at Arlington (protocol 2022-0581). The research involved human participants and adhered to appropriate ethical review and approvals as per institutional guidelines.

#### Informed Consent

Participants were given an online consent form, detailing the study’s purpose, procedures, potential risks, and benefits. They expressed their voluntary agreement to participate by clicking on the “Continue” button on their computer screen. Participants were informed of their ability to opt out of the study at any time without any consequences.

#### Privacy and Confidentiality

All data collected were anonymized to protect the privacy and confidentiality of participants. No personally identifiable information was collected. Data were stored securely on password-protected servers, and only the research team had access to the anonymized data set.

#### Compensation Details

Participants were compensated for their time and effort. Each participant received US $0.25 for completing the survey, which is a standard compensation rate for similar studies conducted on MTurk. This compensation was designed to be fair and transparent, ensuring that participants were adequately reimbursed for their contribution to the research.

## Results

We received 1222 completed surveys (completion rate of 94.5%) from a total of 1293 attempts in December 2022. Participants took an average of 3 minutes to complete the survey. The majority of the participants were younger than 65 years, White, held a bachelor’s degree, reported an income range of US $40,000-80,000, and had 1 or more chronic conditions (Table 2). Detailed comparisons on sex, race, education, income, and number of chronic conditions are reported in the Multimedia Appendix 1.

**Figure 1:**
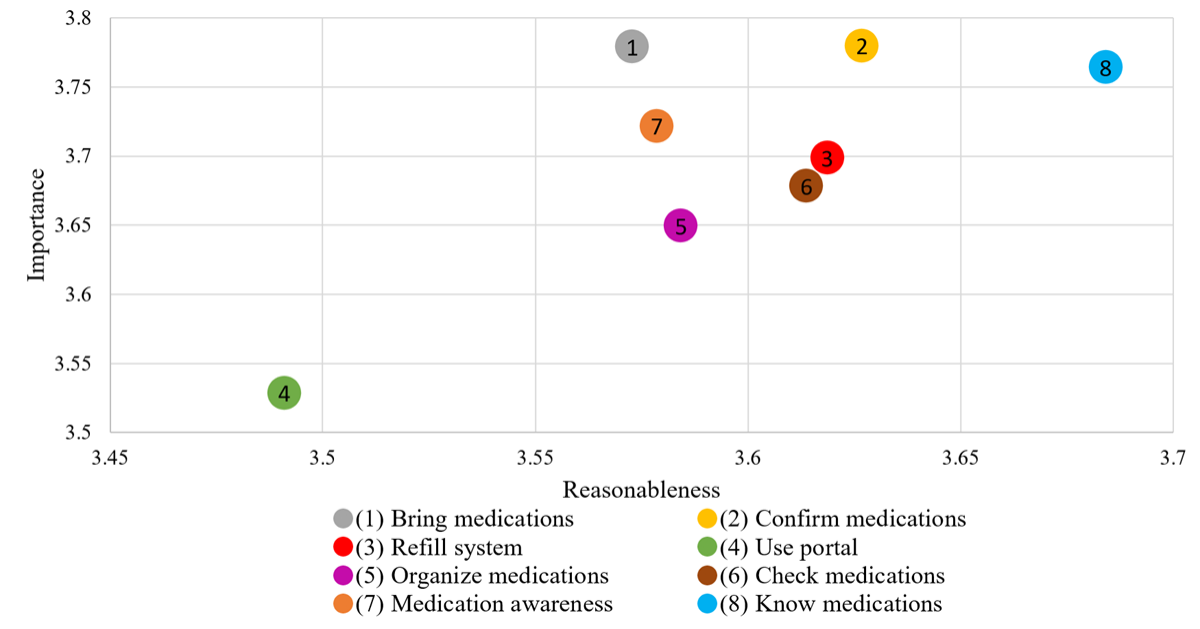
Perceptions of behaviors in terms of importance and reasonableness (n=1222). Range scale was used.

Across age groups, “confirming medications” was rated as the most important behavior while “knowing medications” was rated as the most reasonable behavior (Figure 1). In contrast, the behavior of using portals received the lowest scores for both importance and reasonableness. The perceived importance and reasonableness of each behavior were positively correlated (*P*<.001; correlations in Table S1 in Multimedia Appendix 1). Serious disagreements between importance and reasonableness for the 8 behaviors were between 6% and 7.3% among the participants (Table S2 in Multimedia Appendix 1). In addition, older adult participants reported higher importance and lower reasonableness ratings across the 8 identified behaviors (Multimedia Appendix 2).

Among older adult participants, the behavior of confirming medication was scored highest in importance, whereas the younger age participants scored the behavior of bringing medications as highest in importance (Multimedia Appendix 3). Furthermore, among the older adult participants, the behaviors of getting help (*P*=.007) and knowing medications (*P*=.03) were rated as the second and third most important, respectively, and were found to be significantly higher than those rated by younger adult participants.

The behavior of using portals scored lowest in both reasonableness and importance for both age groups. In addition, there was no significant difference in the scoring of this behavior between the 2 age groups. Out of the total, 3 of the remaining behaviors were scored significantly higher by older adult participants than younger adult participants (Multimedia Appendix 3).

Across different age groups, we observed a general negative association between the perceived importance and reasonableness of these behaviors with male, Hispanic or Latino ethnicity, and education levels. Conversely, higher income was associated with a positive perception of importance. In addition, chronic medical conditions were linked to a negative perception of reasonableness. A detailed breakdown of these findings by individual behavior is shown in the Multimedia Appendix 1.

**Table 2:** Demographics of study participants (n=1222).

Characteristic		<65 years	≥65 years	Proportion test across age groups, *P* value
Age (years), mean (SD)		36.77 (10.73)	68.70 (2.95)	—^a^
Female sex, n (%)		497 (45.31)	64 (51.2)	.21
**Race, n (%)**				
	White	934 (85.1)	83 (66.4)	<.001
	Non-White	163 (14.9)	42 (33.6)	<.001
Hispanic or Latino ethnicity, n (%)		327 (29.8)	40 (32)	.61
**Education, n (%)**				
	Less than bachelor’s degree	131 (11.9)	42 (33.6)	<.001
	Bachelor’s degree	679 (61.9)	45 (36)	<.001
	Graduate degree	287 (26.2)	38 (30.4)	.31
**Annual household income (US $), n (%)**				
	<40,000	300 (27.3)	44 (35.2)	.07
	40,000-80,000	659 (60.1)	70 (56)	.38
	>80,000	138 (12.6)	11 (8.8)	.22
**Chronic medical conditions, n (%)**				
	0	418 (38.1)	24 (19.2)	<.001
	≥1	679 (61.9)	101 (80.8)	<.001

^a^—: not applicable.

## Discussion

Our study found that older adults perceive a higher importance and reasonableness of medication safety behaviors compared with younger adults. Specifically, confirming medications and knowing medications were rated as the most important and reasonable behaviors across age groups while using patient portals was perceived as the least important and reasonable.

Previous research and safety advocacy organizations have suggested behaviors that patients can adopt to improve medication safety in ambulatory settings. Figure 1 provides an overview of participants’ perceptions of each behavior in terms of importance and reasonableness. Despite advocacy from policy makers and professional organizations for patient portal usage [6,42], the results show that it is perceived as the least reasonable and important behavior across age groups. This could be due to a perceived disconnection between portals and medication-related safety, although our study did not directly explore this link. It is important to note that the survey question about patient portal usage did not explicitly link its use to performing other medication safety behaviors, which may have influenced participants’ perceptions. Nonetheless, the discrepancy highlights the importance of understanding patients’ perspectives in shaping policy and clinical practices.

Conversely, the behaviors of confirming medications and knowing medications received the highest scores in terms of both importance and reasonableness. These findings emphasize the significance of these behaviors in patient engagement and medication safety efforts. Health care providers should recognize the value placed by patients on these aspects of medication management and incorporate discussions and interventions related to these behaviors into clinical practice.

Our analysis also revealed a strong correlation between reasonableness and importance for all behaviors among participants. This suggests that intervention strategies may consider targeting efforts to explain the importance and values of these behaviors. This aligns with a cost-benefit thinking approach, where something is considered reasonable when its benefits (ie, importance) are perceived as higher than the efforts required [43]. By emphasizing the benefits of engaging in medication safety practices, health care providers can encourage patients to adopt these behaviors more effectively.

Our results suggested that older adults may be more cognizant of and experienced with health issues, making them more willing to expend effort to carry out the identified behaviors. Further research is needed to understand the reasons behind this observed difference in scores between the 2 age groups.

Our study has several limitations. While role-playing experiments are widely used in marketing science and health care [10,44], the online platform used in the study, MTurk, may introduce biases in the sample population [45]. Consequently, the perspectives of our participants may not fully represent the broader demographic, particularly older adults, in terms of sex, race, education level, and chronic conditions. Furthermore, the underrepresentation of participants older than 65 years (10% vs 17% in the US population) could limit the generalizability of our findings. However, we mitigated this by analyzing participants older than 65 years separately from those younger than 65 years, allowing us to explore differences in perceptions of medication safety behaviors between these distinct age groups.

In addition, it is important to recognize the complexity inherent in assessing perceptions of importance, particularly across diverse demographic groups. While our study aimed to capture patient perspectives on medication safety behaviors, the construct of importance is multifaceted and may be influenced by individual experiences, beliefs, and priorities. This highlights the potential for bias in responses when using a single age frame, particularly for younger participants who might underestimate the capabilities and perspectives of older adults.

Furthermore, the survey question about patient portal usage did not explicitly link its use to performing other medication safety behaviors, which may have influenced participants’ perceptions. The lack of context regarding the comprehensive use of patient portals could have impacted the ratings given by participants, thus presenting a limitation in accurately assessing the perceived importance and reasonableness of using patient portals.

Future research should consider using alternative sampling methods, such as stratified sampling or oversampling of underrepresented groups, to ensure greater representativeness in the sample. Conducting similar studies with a purposive sampling strategy could provide a more comprehensive understanding of patient perceptions of medication safety behaviors across diverse demographic groups, including older adults with multiple chronic conditions. Given the study’s limited scope, qualitative components were not integrated into the survey. Subsequent studies should encompass qualitative interviews with a spectrum of individuals, both laypersons and professionals. Furthermore, careful consideration should be given to the age-related framing of survey questions. Using age-specific framing tailored to different groups, such as presenting scenarios relevant to their age experiences, could minimize bias and provide more accurate insights into participant perceptions and behaviors across various age ranges, ultimately leading to findings with greater validity and generalizability.

### Conclusion

Our study found that older adults perceive higher importance of a set of safety medication behaviors and see these behaviors as more reasonable to perform than younger adults. Using portals is generally perceived lower in importance and reasonableness by patients in ambulatory settings when compared with other medication safety behaviors, such as bringing medications to clinic visits. Future studies should explore additional factors influencing patient engagement in medication safety behaviors including social determinants of health. Longitudinal studies are needed to understand how improvement efforts can take advantage of patient perspectives on medication safety to design interventions to encourage the adoption of specific behaviors.

## Appendix

Multimedia Appendix 1 Mturk Experimental Instructions.

Multimedia Appendix 2 Perceptions of combined behaviors in terms of importance and reasonableness among age groups (n=1097 for age <65 years and n=125 for age ≥65 years).

Multimedia Appendix 3 Perceptions of behaviors in terms of importance and reasonableness among age groups (n=1097 for age <65 years and n=125 for age ≥65 years). Wilcoxon rank-sum test *P*<.001.

## Abbreviations

AHRQ: Agency for Healthcare Research and Quality
IRB: institutional review board
MTurk: Amazon Mechanical Turk
PROMIS: Partnership for Resilience in Medication Safety
SoPHIE: Software Platform for Human Interaction Experiments

## Data Availability

All data generated or analyzed during this study are included in this published paper and its supplementary information files.
