# Supplementary material for: Perception of Medication Safety–Related Behaviors Among Different Age Groups: Web-Based Cross-Sectional Study
Source: Interact J Med Res. 2024 Aug 12;13:e58635. doi: 10.2196/58635 (PMC11347903; doi:10.2196/58635)
Supplement: Multimedia Appendix 1 [file ijmr_v13i1e58635_app1.docx]

**Table S1.** Pearson correlation coefficient of importance and reasonableness for each behavior

| Correlation of Importance and Reasonableness | | |
| --- | --- | --- |
|  | coefficient | *p* value |
| Bring medications | 0.543 | 0.001 |
| Confirm medications | 0.511 | 0.001 |
| Refill system | 0.494 | 0.001 |
| Use portal | 0.436 | 0.001 |
| Organize medications | 0.470 | 0.001 |
| Check medications | 0.530 | 0.001 |
| Medication awareness | 0.521 | 0.001 |
| Know medications | 0.487 | 0.001 |

**Table S2.** Serious disagreement (count) of importance and reasonableness for each behavior

|  | 1 vs 4, 2 vs 5 and 1 vs 5 | | 4 vs 1, 5 vs 2 and 5 vs 1 | |
| --- | --- | --- | --- | --- |
| Behaviors | n | % | n | % |
| Bring medications | 34 | 2.78% | 50 | 4.09% |
| Confirm medications | 29 | 2.37% | 44 | 3.60% |
| Refill system | 37 | 3.03% | 40 | 3.27% |
| Use portal | 39 | 3.19% | 46 | 3.76% |
| Organize medications | 43 | 3.52% | 46 | 3.76% |
| Check medications | 46 | 3.76% | 43 | 3.52% |
| Medication awareness | 35 | 2.86% | 38 | 3.11% |
| Know medications | 39 | 3.19% | 39 | 3.19% |

**Table S3.** Wilcoxon test of importance or reasonableness (*p* value) by behavior

| Reasonableness | Bring medications | Confirm medications | Manage refills | Use portals | Organize medications | Check medications | Get help |
| --- | --- | --- | --- | --- | --- | --- | --- |
| Confirm medications | 0.921 |  |  |  |  |  |  |
| Manage refills | 0.720 | 0.593 |  |  |  |  |  |
| Use portals | 0.005 | 0.001 | 0.001 |  |  |  |  |
| Organize medications | 0.573 | 0.189 | 0.514 | 0.006 |  |  |  |
| Check medications | 0.549 | 0.775 | 0.580 | 0.001 | 0.327 |  |  |
| Get help | 0.428 | 0.128 | 0.257 | 0.008 | 0.978 | 0.180 |  |
| Know medications | 0.026 | 0.158 | 0.063 | 0.001 | 0.005 | 0.208 | 0.001 |
|  | | | | | | | |
| Important | Bring medications | Confirm medications | Manage refills | Use portals | Organize medications | Check medications | Get help |
| Confirm medications | 0.387 |  |  |  |  |  |  |
| Manage refills | 0.004 | 0.015 |  |  |  |  |  |
| Use portals | 0.001 | 0.001 | 0.001 |  |  |  |  |
| Organize medications | 0.001 | 0.001 | 0.162 | 0.001 |  |  |  |
| Check medications | 0.007 | 0.023 | 0.879 | 0.001 | 0.232 |  |  |
| Get help | 0.019 | 0.027 | 0.458 | 0.001 | 0.038 | 0.280 |  |
| Know medications | 0.273 | 0.420 | 0.084 | 0.001 | 0.001 | 0.082 | 0.359 |

**Table S4.** Wilcoxon signed-rank test compared each behavior (*p* value) among age groups

| Reasonableness (Age < 65) | Bring medications | Confirm medications | Manage refills | Use portals | Organize medications | Check medications | Get help |
| --- | --- | --- | --- | --- | --- | --- | --- |
| Confirm medications | 0.700 |  |  |  |  |  |  |
| Manage refills | 0.468 | 0.634 |  |  |  |  |  |
| Use portals | 0.012 | 0.003 | 0.012 |  |  |  |  |
| Organize medications | 0.352 | 0.219 | 0.641 | 0.042 |  |  |  |
| Check medications | 0.611 | 0.934 | 0.447 | 0.001 | 0.276 |  |  |
| Get help | 0.420 | 0.276 | 0.401 | 0.029 | 0.894 | 0.188 |  |
| Know medications | 0.074 | 0.178 | 0.061 | 0.001 | 0.007 | 0.344 | 0.003 |
|  | | | | | | | |
| Important (Age < 65) | Bring medications | Confirm medications | Manage refills | Use portals | Organize medications | Check medications | Get help |
| Confirm medications | 0.115 |  |  |  |  |  |  |
| Manage refills | 0.002 | 0.079 |  |  |  |  |  |
| Use portals | 0.001 | 0.001 | 0.001 |  |  |  |  |
| Organize medications | 0.001 | 0.009 | 0.252 | 0.007 |  |  |  |
| Check medications | 0.003 | 0.059 | 0.993 | 0.001 | 0.437 |  |  |
| Get help | 0.004 | 0.047 | 0.608 | 0.001 | 0.123 | 0.358 |  |
| Know medications | 0.119 | 0.657 | 0.125 | 0.001 | 0.006 | 0.104 | 0.334 |
|  | | | | | | | |
| Reasonableness (Age ≥ 65) | Bring medications | Confirm medications | Manage refills | Use portals | Organize medications | Check medications | Get help |
| Confirm medications | 0.123 |  |  |  |  |  |  |
| Manage refills | 0.293 | 0.759 |  |  |  |  |  |
| Use portals | 0.176 | 0.003 | 0.009 |  |  |  |  |
| Organize medications | 0.298 | 0.650 | 0.496 | 0.009 |  |  |  |
| Check medications | 0.720 | 0.237 | 0.613 | 0.058 | 0.880 |  |  |
| Get help | 0.914 | 0.092 | 0.276 | 0.053 | 0.635 | 0.776 |  |
| Know medications | 0.095 | 0.684 | 0.801 | 0.001 | 0.424 | 0.251 | 0.109 |
|  | | | | | | | |
| Important (Age ≥ 65) | Bring medications | Confirm medications | Manage refills | Use portals | Organize medications | Check medications | Get help |
| Confirm medications | 0.030 |  |  |  |  |  |  |
| Manage refills | 0.829 | 0.008 |  |  |  |  |  |
| Use portals | 0.011 | 0.001 | 0.001 |  |  |  |  |
| Organize medications | 0.244 | 0.002 | 0.319 | 0.045 |  |  |  |
| Check medications | 0.684 | 0.098 | 0.624 | 0.003 | 0.131 |  |  |
| Get help | 0.131 | 0.274 | 0.408 | 0.001 | 0.049 | 0.485 |  |
| Know medications | 0.198 | 0.181 | 0.397 | 0.001 | 0.034 | 0.522 | 0.966 |

**Table S5.** Pearson correlation coefficient of importance and reasonableness for each behavior among age groups

| Correlation of importance and reasonableness | age < 65 | | age ≥ 65 | |
| --- | --- | --- | --- | --- |
|  | coefficient | *p* value | coefficient | *p* value |
| Bring medications | 0.530 | 0.001 | 0.648 | 0.001 |
| Confirm medications | 0.493 | 0.001 | 0.663 | 0.001 |
| Refill system | 0.472 | 0.001 | 0.661 | 0.001 |
| Use portal | 0.418 | 0.001 | 0.604 | 0.001 |
| Organize medications | 0.452 | 0.001 | 0.632 | 0.001 |
| Check medications | 0.517 | 0.001 | 0.644 | 0.001 |
| Medication awareness | 0.502 | 0.001 | 0.681 | 0.001 |
| Know medications | 0.477 | 0.001 | 0.564 | 0.001 |

**Table S6.** Perceptions of behaviors in terms of importance and reasonableness (*n* = 1,222), ranked in descending order.

|  | Importance |  | Reasonableness |
| --- | --- | --- | --- |
| Behaviors | Mean (±SD) | Behaviors | Mean (±SD) |
| Confirm medications | 3.78 (1.26) | Know medications | 3.68 (1.27) |
| Bring medications | 3.78 (1.34) | Confirm medications | 3.63 (1.27) |
| Know medications | 3.76 (1.25) | Manage refills | 3.62 (1.25) |
| Medication awareness | 3.72 (1.29) | Check medications | 3.61 (1.33) |
| Refill system | 3.70 (1.29) | Organize medications | 3.58 (1.26) |
| Confirm medications | 3.68 (1.35) | Get help | 3.58 (1.26) |
| Organize medications | 3.65 (1.27) | Bring medications | 3.57 (1.37) |
| Use portals | 3.53 (1.25) | Use portals | 3.49 (1.26) |

Note: Responses are on a 5-point Likert scale.

**Table S7.** Perceptions of behaviors in terms of importance and reasonableness among age groups (*n*=1,097 and 125 for age <65 and ≥65, respectively)

|  | Importance | | Reasonableness | |
| --- | --- | --- | --- | --- |
| Age Groups | < 65 | ≥ 65 | < 65 | ≥ 65 |
| Behaviors | Mean (±SD) | Mean (±SD) | Mean (±SD) | Mean (±SD) |
| Bring medications | 3.77 (1.33) | 3.82 (1.36) | 3.56 (1.36) | 3.72 (1.38) |
| Confirm medications | 3.75 (1.26)* | 4.07 (1.22) | 3.59 (1.27)* | 3.94 (1.18) |
| Refill system | 3.68 (1.29) | 3.87 (1.30) | 3.58 (1.25)* | 3.94 (1.26) |
| Use portal | 3.53 (1.25) | 3.52 (1.26) | 3.48 (1.26) | 3.59 (1.21) |
| Organize medications | 3.64 (1.26) | 3.76 (1.33) | 3.55 (1.27)* | 3.88 (1.18) |
| Check medications | 3.65 (1.36) | 3.89 (1.23) | 3.59 (1.32) | 3.80 (1.34) |
| Medication awareness | 3.69 (1.28)* | 3.98 (1.28) | 3.55 (1.26)* | 3.79 (1.21) |
| Know medications | 3.74 (1.26)* | 3.98 (1.18) | 3.65 (1.27)* | 3.98 (1.19) |

***:** Wilcoxon rank sum test *p* < 0.05.

**Table S8. Subgroup analyses for variables including gender, race, ethnicity, education, household income, and chronic medical conditions (Ordered logistic regression).**

| 1. Gender | Reasonableness | | | | Importantce | | | |
| --- | --- | --- | --- | --- | --- | --- | --- | --- |
|  | Age_group | | Gender | | Age_group | | Gender | |
|  | coeff | p-value | coeff | p-value | coeff | p-value | coeff | p-value |
| Overall | 0.538 | 0.001 | -0.260 | 0.009 | 0.408 | 0.014 | -0.304 | 0.002 |
| Bring medications | 0.265 | 0.125 | 0.016 | 0.874 | 0.089 | 0.610 | -0.112 | 0.284 |
| Confirm medications | 0.510 | 0.003 | -0.295 | 0.004 | 0.554 | 0.002 | -0.344 | 0.001 |
| Refill system | 0.612 | 0.001 | -0.280 | 0.007 | 0.318 | 0.067 | -0.183 | 0.076 |
| Use portal | 0.143 | 0.389 | -0.128 | 0.213 | -0.029 | 0.865 | -0.140 | 0.174 |
| Organize medications | 0.486 | 0.005 | -0.196 | 0.057 | 0.229 | 0.191 | -0.228 | 0.027 |
| Check medications | 0.329 | 0.060 | -0.226 | 0.028 | 0.251 | 0.141 | -0.369 | 0.000 |
| Medication awareness | 0.341 | 0.044 | -0.162 | 0.115 | 0.479 | 0.007 | -0.283 | 0.007 |
| Know medications | 0.483 | 0.006 | -0.314 | 0.002 | 0.346 | 0.044 | -0.207 | 0.046 |
| Note: Age_group = 0 if age < 65; Gender = 0 if Female. | | | |  |  |  |  |  |
|  |  |  |  |  |  |  |  |  |
| 2. Race | Reasonableness | | | | Importantce | | | |
|  | Age_group | | Race | | Age_group | | Race | |
|  | coeff | p-value | coeff | p-value | coeff | p-value | coeff | p-value |
| Overall | 0.575 | 0.000 | -0.156 | 0.257 | 0.419 | 0.013 | -0.009 | 0.949 |
| Bring medications | 0.295 | 0.092 | -0.142 | 0.316 | 0.112 | 0.522 | -0.114 | 0.425 |
| Confirm medications | 0.529 | 0.003 | -0.064 | 0.657 | 0.570 | 0.002 | -0.036 | 0.802 |
| Refill system | 0.589 | 0.001 | 0.166 | 0.253 | 0.329 | 0.062 | -0.012 | 0.935 |
| Use portal | 0.175 | 0.298 | -0.143 | 0.306 | -0.006 | 0.973 | -0.072 | 0.610 |
| Organize medications | 0.520 | 0.003 | -0.173 | 0.220 | 0.229 | 0.199 | 0.057 | 0.689 |
| Check medications | 0.365 | 0.038 | -0.114 | 0.412 | 0.280 | 0.104 | 0.000 | 0.998 |
| Medication awareness | 0.353 | 0.040 | -0.025 | 0.861 | 0.470 | 0.009 | 0.094 | 0.508 |
| Know medications | 0.509 | 0.004 | -0.037 | 0.794 | 0.335 | 0.054 | 0.121 | 0.402 |
| Note: Age_group = 0 if age < 65; Race = 0 if White. | | | |  |  | 0.054 |  |  |
|  |  |  |  |  |  |  |  |  |
| 3. Ethnicity | Reasonableness | | | | Importantce | | | |
|  | Age_group | | Ethnicity | | Age_group | | Ethnicity | |
|  | coeff | p-value | coeff | p-value | coeff | p-value | coeff | p-value |
| Overall | 0.573 | 0.000 | -0.530 | 0.000 | 0.448 | 0.007 | -0.652 | 0.000 |
| Bring medications | 0.282 | 0.103 | -0.306 | 0.007 | 0.109 | 0.530 | -0.397 | 0.001 |
| Confirm medications | 0.528 | 0.002 | -0.456 | 0.000 | 0.583 | 0.001 | -0.592 | 0.000 |
| Refill system | 0.627 | 0.000 | -0.269 | 0.018 | 0.358 | 0.040 | -0.496 | 0.000 |
| Use portal | 0.155 | 0.353 | -0.214 | 0.057 | -0.013 | 0.939 | -0.287 | 0.011 |
| Organize medications | 0.493 | 0.004 | -0.289 | 0.011 | 0.258 | 0.142 | -0.545 | 0.000 |
| Check medications | 0.348 | 0.046 | -0.431 | 0.000 | 0.316 | 0.063 | -0.454 | 0.000 |
| Medication awareness | 0.374 | 0.028 | -0.492 | 0.000 | 0.507 | 0.004 | -0.453 | 0.000 |
| Know medications | 0.533 | 0.002 | -0.463 | 0.000 | 0.385 | 0.025 | -0.592 | 0.000 |
| Note: Age_group = 0 if age < 65; Ethnicity = 0 if Non-Hispanic or Non-Latino. | | | | | |  |  |  |
|  |  |  |  |  |  |  |  |  |
| 4. Education | Reasonableness | | | | Importantce | | | |
|  | Age_group | | Education | | Age_group | | Education | |
|  | coeff | p-value | coeff | p-value | coeff | p-value | coeff | p-value |
| Overall | 0.520 | 0.002 | -0.124 | 0.036 | 0.373 | 0.025 | -0.158 | 0.008 |
| Bring medications | 0.226 | 0.194 | -0.196 | 0.002 | 0.026 | 0.880 | -0.229 | 0.000 |
| Confirm medications | 0.469 | 0.007 | -0.197 | 0.001 | 0.514 | 0.004 | -0.206 | 0.001 |
| Refill system | 0.576 | 0.001 | -0.170 | 0.006 | 0.296 | 0.090 | -0.114 | 0.062 |
| Use portal | 0.132 | 0.429 | -0.079 | 0.185 | -0.043 | 0.799 | -0.082 | 0.180 |
| Organize medications | 0.466 | 0.007 | -0.079 | 0.189 | 0.217 | 0.218 | -0.079 | 0.194 |
| Check medications | 0.345 | 0.049 | -0.001 | 0.990 | 0.277 | 0.105 | -0.011 | 0.857 |
| Medication awareness | 0.332 | 0.051 | -0.061 | 0.317 | 0.447 | 0.012 | -0.165 | 0.008 |
| Know medications | 0.482 | 0.006 | -0.094 | 0.123 | 0.309 | 0.072 | -0.193 | 0.002 |
| Note: Age_group = 0 if age < 65; Education = 0 if less than college degress; Education = 1 if college degress; Education = 2 if graduate degress. | | | | | | | | |
|  |  |  |  |  |  |  |  |  |
| 5. Income | Reasonableness | | | | Importantce | | | |
|  | Age_group | | Income | | Age_group | | Income | |
|  | coeff | p-value | coeff | p-value | coeff | p-value | coeff | p-value |
| Overall | 0.565 | 0.001 | 0.045 | 0.069 | 0.453 | 0.007 | 0.085 | 0.001 |
| Bring medications | 0.269 | 0.120 | 0.021 | 0.412 | 0.105 | 0.546 | 0.071 | 0.006 |
| Confirm medications | 0.532 | 0.002 | 0.062 | 0.015 | 0.575 | 0.001 | 0.064 | 0.013 |
| Refill system | 0.633 | 0.000 | 0.051 | 0.044 | 0.346 | 0.047 | 0.055 | 0.030 |
| Use portal | 0.157 | 0.347 | 0.032 | 0.208 | -0.004 | 0.982 | 0.062 | 0.014 |
| Organize medications | 0.496 | 0.004 | 0.034 | 0.178 | 0.260 | 0.139 | 0.073 | 0.004 |
| Check medications | 0.347 | 0.047 | 0.008 | 0.743 | 0.295 | 0.084 | 0.063 | 0.015 |
| Medication awareness | 0.365 | 0.032 | 0.062 | 0.015 | 0.504 | 0.005 | 0.063 | 0.014 |
| Know medications | 0.518 | 0.003 | 0.057 | 0.025 | 0.377 | 0.028 | 0.065 | 0.011 |
| Note: Age_group = 0 if age < 65; Income = 0 if < $40,000; Income = 1 if $40,000 - $80,000; Income = 2 if > $80,000. | | | | | | | | |
|  |  |  |  |  |  |  |  |  |
| 6. Chronic medical conditions | Reasonableness | | | | Importantce | | | |
|  | Age_group | | Chronic | | Age_group | | Chronic | |
|  | coeff | p-value | coeff | p-value | coeff | p-value | coeff | p-value |
| Overall | 0.593 | 0.000 | -0.223 | 0.034 | 0.440 | 0.009 | -0.113 | 0.277 |
| Bring medications | 0.288 | 0.097 | -0.116 | 0.283 | 0.079 | 0.653 | 0.071 | 0.516 |
| Confirm medications | 0.588 | 0.001 | -0.364 | 0.001 | 0.614 | 0.001 | -0.244 | 0.025 |
| Refill system | 0.633 | 0.000 | -0.075 | 0.486 | 0.368 | 0.035 | -0.208 | 0.055 |
| Use portal | 0.167 | 0.320 | -0.090 | 0.404 | -0.037 | 0.829 | 0.081 | 0.449 |
| Organize medications | 0.501 | 0.004 | -0.072 | 0.500 | 0.218 | 0.218 | 0.113 | 0.294 |
| Check medications | 0.411 | 0.019 | -0.324 | 0.003 | 0.339 | 0.048 | -0.272 | 0.013 |
| Medication awareness | 0.379 | 0.027 | -0.160 | 0.138 | 0.513 | 0.004 | -0.124 | 0.252 |
| Know medications | 0.536 | 0.002 | -0.165 | 0.126 | 0.398 | 0.021 | -0.209 | 0.055 |
| Note: Age_group = 0 if age < 65; Chronic = 0 if 0 chronic medical conditions. | | | | | |  |  |  |

**Table S9. Analyses for three age groups.**

|  | Importantce | | | Reasonableness | | |
| --- | --- | --- | --- | --- | --- | --- |
|  | Age < 40 | 40 ≤ Age < 65 | Age ≥ 65 | Age < 40 | 40 ≤ Age < 65 | Age ≥ 65 |
| Overall | 3.59 | 3.86 | 3.86 | 3.47 | 3.75 | 3.83 |
| Bring medications | 3.65 | 3.99 | 3.82 | 3.45 | 3.74 | 3.72 |
| Confirm medications | 3.67 | 3.89 | 4.07 | 3.52 | 3.72 | 3.94 |
| Refill system | 3.58 | 3.86 | 3.87 | 3.45 | 3.82 | 3.94 |
| Use portal | 3.48 | 3.63 | 3.52 | 3.43 | 3.56 | 3.59 |
| Organize medications | 3.58 | 3.74 | 3.76 | 3.44 | 3.76 | 3.88 |
| Check medications | 3.52 | 3.90 | 3.89 | 3.48 | 3.80 | 3.80 |
| Medication awareness | 3.56 | 3.94 | 3.98 | 3.48 | 3.69 | 3.79 |
| Know medications | 3.65 | 3.91 | 3.98 | 3.53 | 3.87 | 3.98 |

**Figure S1.** MTurk instructions to the subject

**
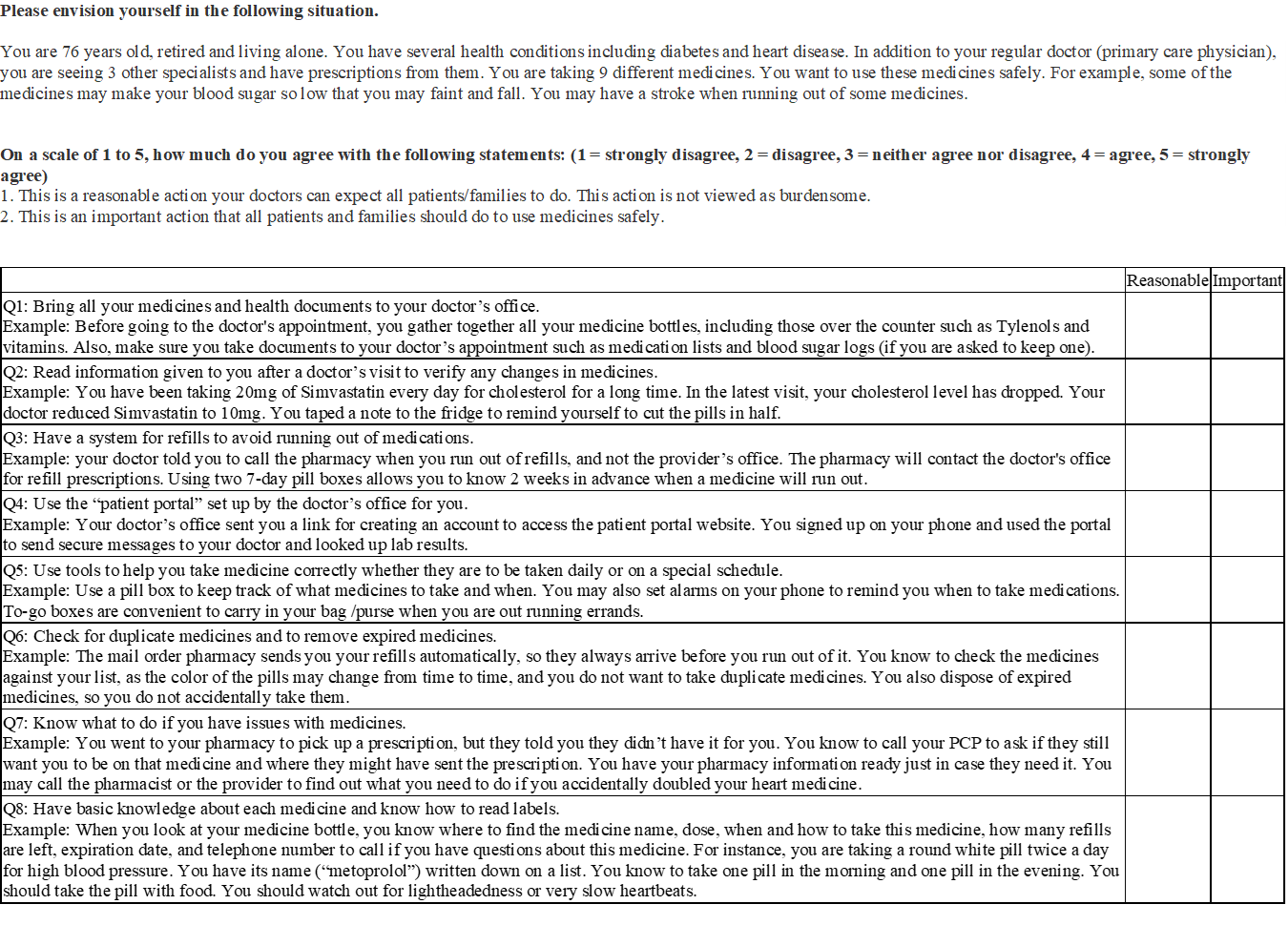
**
