# Supplementary figures and images for: Perception of Medication Safety–Related Behaviors Among Different Age Groups: Web-Based Cross-Sectional Study
Source: Interact J Med Res. 2024 Aug 12;13:e58635. doi: 10.2196/58635 (PMC11347903; doi:10.2196/58635)

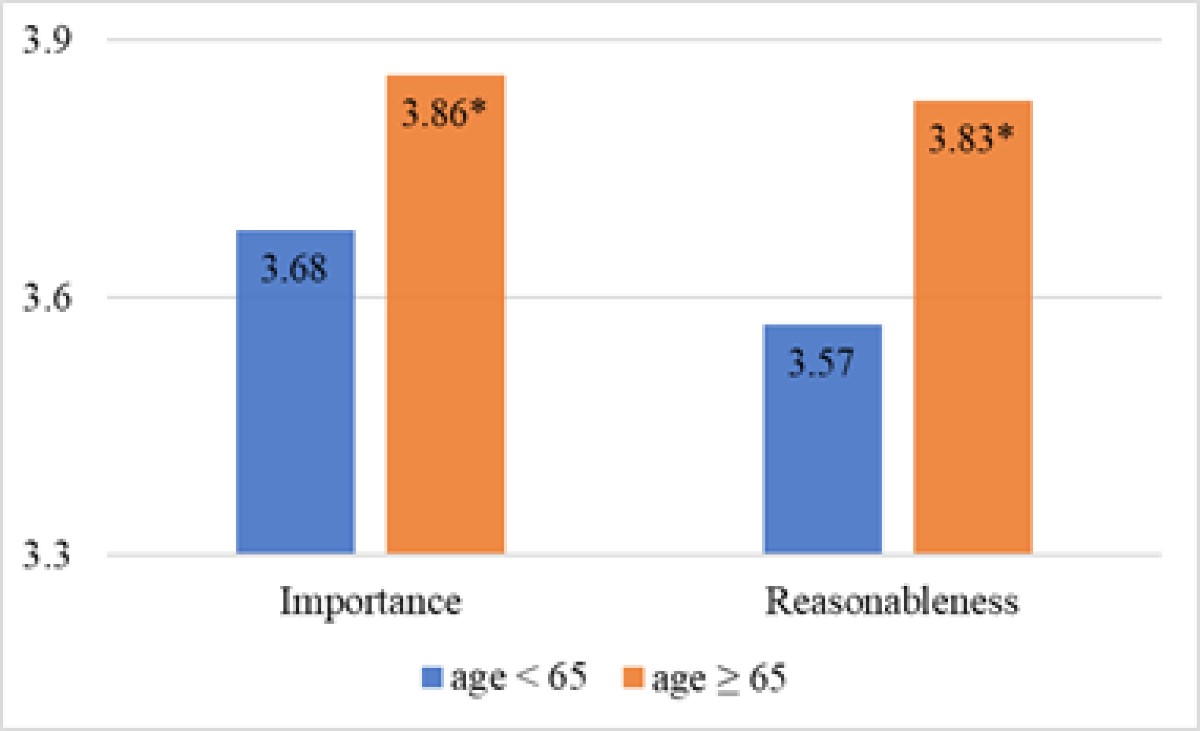


* Wilcoxon rank-sum test *P*<.001.

Supplement: Multimedia Appendix 2 [file ijmr_v13i1e58635_app2.docx]

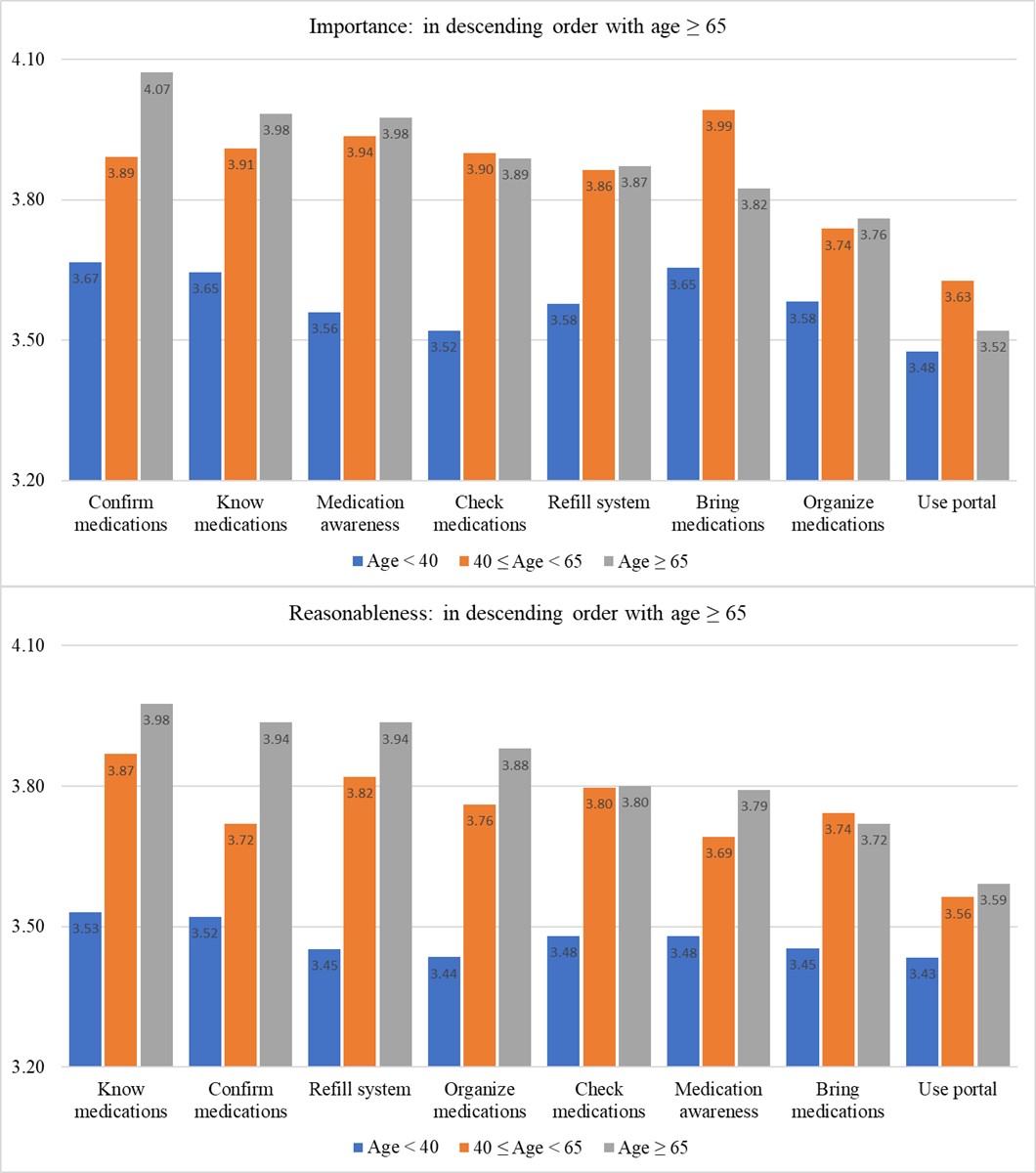

Supplement: Multimedia Appendix 3 [file ijmr_v13i1e58635_app3.docx]
